# Breathable Films with Self-Cleaning and Antibacterial Surfaces Based on TiO_2_-Functionalized PET Membranes

**DOI:** 10.3390/membranes13080733

**Published:** 2023-08-15

**Authors:** Olga Alisiyonak, Anna Lavitskaya, Liudmila Khoroshko, Artem L. Kozlovskiy, Maxim Zdorovets, Ilya Korolkov, Maryia Yauseichuk, Egor Kaniukov, Alena Shumskaya

**Affiliations:** 1 Faculty of Chemical Technology and Engineering, Belarusian State Technological University, 13a Sverdlova Str., 220006 Minsk, Belarus; 2 Faculty of Physics, Belarusian State University, 4 Nezavisimosti Av., 220030 Minsk, Belarus; 3 R&D Department, Belarusian State University of Informatics and Radioelectronics, 6 P. Browka Str., 220013 Minsk, Belarus; 4 Engineering Profile Laboratory, Gumilyov Eurasian National University, 11 Satpaeva Str., Nur-Sultan 010000, Kazakhstan; 5 The Institute of Nuclear Physics, 1 Ibragimova Str., Almaty 050032, Kazakhstan; 6 Department of Materials Technology of Electronics, National University of Science and Technology, “MISIS”, Leninsky Av. 4, Moscow 119049, Russia; ka.egor@mail.ru; 7 Institute of Chemistry of New Materials, 36 F. Skaryna Str., 220141 Minsk, Belarus

**Keywords:** PET membrane, titanium dioxide, photocatalysis, packing technology, logarithmic reduction factors

## Abstract

A promising approach that uses the sol–gel method to manufacture new breathable active films with self-cleaning and antibacterial surfaces is based on the PET membranes obtained via ion track technology with a pore density of 10^–7^ cm^−2^ and a pore diameter of about 500 ± 15 nm, coated with a layer of TiO_2_ anatase, with a thickness of up to 80 nm. The formation of the photocatalytically active TiO_2_ anatase phase was confirmed using Raman analysis. Coating the PET membrane with a layer of TiO_2_ increased the hydrophobicity of the system (CA increased from 64.2 to 92.4, and the antibacterial activity was evaluated using *Escherichia coli* and *Staphylococcus aureus* bacteria with the logarithmic reduction factors of 3.34 and 4.24, respectively).

## 1. Introduction

The effect of self-cleaning surfaces created with functional coatings can be used in a wide range of applications, from household paints to the functional coatings of microelectronics for space applications [1,2,3]. Due to their low costs, well-adapted production technology, physical and chemical high stability, and low toxicity, TiO_2_ and ZnO are the most common additives used for self-cleaning and antibacterial surfaces [4,5]. Titanium-dioxide-based structures are widely used as sorbents and catalysts. TiO_2_ photocatalysts can be activated via UV radiation and visible light and can decompose a large number of organic compounds. For water and air purification purposes, titania can be used in powder form or as film composites on substrates, which contributes to the expansion of photocatalyst applications [6,7,8]. Under irradiation, electron–hole pairs form in TiO_2_, and then charge carriers come to the particle surface, migrate over the surface, and may take part in chemical reactions. This leads to the formation of free radicals that can oxidize (and mineralize) almost any organic compound to CO_2_ and H_2_O.

The low production price and photocatalytic activity of TiO_2_ make it an interesting material for purifying water or air from organic compounds. The most effective practical application is the use of TiO_2_ as a coating applied to a structured or porous matrix [9]. The thin-layer coating of a porous structure significantly increases the working surface of the active substance and contributes to the intensification of the process. Surface morphology and chemical composition (for example, any additives or binders) are important for the photoactivity and superhydrophobic properties of TiO_2_ films. Roughness and porous structure will increase the working surface, ensuring the self-cleaning films’ effectiveness. Another important aspect regarding coating is the increase in the contact angle of wetting. In this case, molecules of organic substances contained in a stream of water or air will be not so easily adsorbed on the functionalized TiO_2_ surface of the filtration membrane [10], but the absorbed molecules under UV irradiation will be oxidized to carbon dioxide and water or practically destroyed. These degraded organic contaminants will be easily washed off with water due to the hydrophobic properties of the TiO_2_ coating [11]. In addition, the generated free radicals will be detrimental to harmful microorganisms which are resistant to ultraviolet light under normal conditions.

Investigations on TiO_2_ films have been devoted to the synthesis of nanostructured titania in the form of nanoparticles, nanograined films, or the components of combined materials (particularly polymer matrices) [12,13]. Polymer-based films could improve the flexibility of photocatalytic active structure and expand the applications associated with carrying out the complex purification of liquids (such as ultrafiltration or antibacterial effects) [9,14].

As a rule, TiO_2_ thin films are fabricated by sol–gel dipping, including sols with nanostructured titanium dioxide in particle form. This technology is easy to implement and makes it possible to control the microstructure of both thin films and the properties of gels themselves. There are methods used to create titanium dioxide thin films, such as sputtering [15], ion beam deposition [16], and hydrothermal processes [17], but the sol–gel deposition process has a lower cost and is technologically simple, flexible, and easily scalable [18,19].

For a considerable amount of time, various aspects pertaining to the creation of thin titanium dioxide films using the sol–gel method have been studied. In [20], the impacts of the procedures used to create a nanosol and carry out deposition (immersion rate, acid concentration, and drying temperature) on the structural, optical, and photocatalytic properties of TiO_2_ films were studied. In [21,22], the effects of precursors on the microstructure and optical properties of TiO_2_ films were studied. In addition, the formation of thin titanium dioxide films using a binder, which can increase the stability and durability of the functional coating, such as PVA, PMMA, or PEG, was considered [13,23,24,25].

Previously, we obtained satisfactory results for the creation of thin mechanically stable titanium dioxide without the use of a binder polymer on the surface of a track-etched membrane (PET TM) via a sol–gel method with different active component concentrations [9]. Thus, in this study, we propose an approach that can be used to obtain breathable films with a self-cleaning and antibacterial surface based on titanium-dioxide-coated PET membranes (PET TMs) via sol–gel immersion, study their morphology and structural characteristics, and also evaluate their photocatalytic and antibacterial activities.

## 2. Experimental

### 2.1. “PET TM + TiO_2_” Systems Formation

PET films 12 microns thick (Hostaphan^®^, Mitsubishi Polyester Film, Wiesbaden, Germany) were irradiated on cyclotron DC-60 (Astana, Kazakhstan) with Kr ions with an energy of 1.75 MeV/nucleon and fluence 10^–7^ cm^−2^ and then etched in 2.2M NaOH solution at 85 °C 7 min to obtain pores with diameters about 500 ± 15 nm [26,27].

Titania hydrosols were prepared using a two-step method. In the first stage, hydrated titanium dioxide was precipitated from titanium tetrachloride solutions, and the obtained precipitates were filtered and washed until the negative reaction to chlorine ions. In the second stage, the resulting hydrated titanium dioxide precipitate was peptized using monobasic inorganic acids. The radius *R* of sol particles was measured via DLS using the spectrometer (Metertech 8001, Metertech Inc., Taipei City, Taiwan). The optical density *D* and turbidity spectra of the sols were determined in a 10 mm wide cell, wavelength 566 nm. The solid phase containing titanium dioxide in an obtained hydrosol was 0.5 wt.%. The resulting sols had a titania particle size of 15–50 nm, and the stability period of hydrosol was more than 250 h. The pH of the hydrosol was less than 0.6–0.8.

For the production of “PET TM + TiO_2_” systems, membranes were immersed in a sol with TiO_2_ concentration of 0.5 wt.% for 60 s, then washed in distilled water. Three layers of TiO_2_ were formed, and then PET TM with deposited TiO_2_ was annealed at 120 °C for 15 min on air [28].

### 2.2. Morphology Control

The results of the titanium dioxide deposition were controlled by examining the surface and cross-section of the sample “PET TM + TiO_2_” systems via scanning electron microscopy (SEM, JEOL JCM-6000 Plus Neoscope microscope, JEOL, Akishima, Tokyo) and energy dispersive analysis (EDA, JED-2300 Analysis Station at JEOL microscope, JEOL, Akishima, Tokyo).

### 2.3. Structural Analysis and Measurement of Water and Gas Permeability

Structural features of “PET TM + TiO_2_” systems were checked via Raman spectroscopy (Raman, INTEGRA Spectra, NT-MDT, Westzaan, The Netherlands). UV-vis spectra were recorded on Specord-250 BU (Analytik Jena GmbH, Jena, Germany) on Integrating Spheres in a range from 190 to 800 nm with a scan rate of 10 nm/s.

The air and water permeability were investigated to confirm the breathable properties of “PET TM + TiO_2_” systems [27]. The equipment for the porous diameter measurements via the manometric method for determining gas permeability was used for air permeability tests. The discharge gas pressure in the primary chamber in the 4–20 kPa range and water pressure in the 12–120 kPa range were applied. The membrane was considered permeable if air intake could be registered in the receiving chamber at pressures less than or about equal to atmosphere pressure.

### 2.4. Surface Adsorption Properties Control

The surface adsorption properties of the original and modified samples were evaluated via the contact angle of water wetting (CA) with a volume of a drop of 10 μL. An image of a lying liquid droplet was captured using a camera (resolution 100 × 100 dpi) at 100× magnification. Determination of CA of the samples from the images was carried out in the ImageJ program (https://imagej.nih.gov/ij/download.html, 10 August 2023), averaging the bases of 5 measurements.

### 2.5. Analysis of Photocatalytic Activity

**Figure 1:**
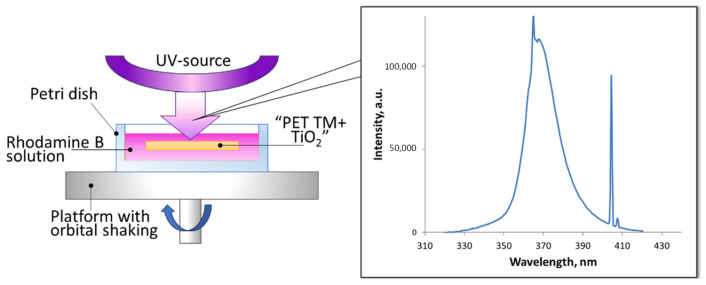
Scheme of the photocatalytic test and the emission spectrum of mercury lamp (on insertion).

**Figure 2:**
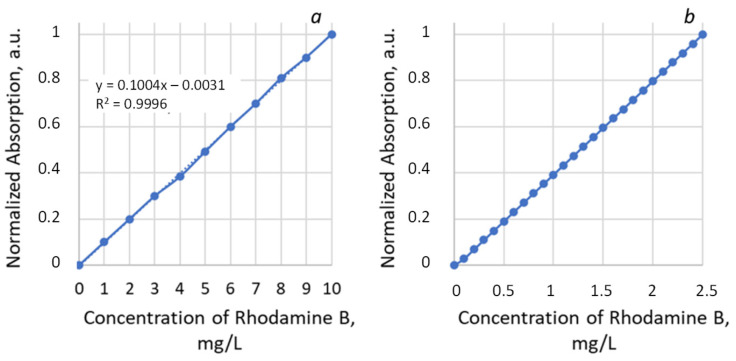
Normalized calibration curve for the concentration range 0–10 mg/L of Rhodamine B with approximation equation (**a**) and reconstructed using equation detailed part for the concentration range 0–2.5 mg/L (**b**).

**Figure 3:**
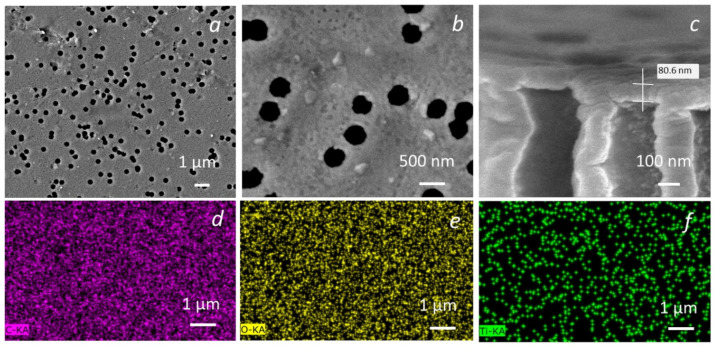
(color online) Morphological and structural investigation of the “PET TM + TiO_2_” systems. SEM of the “PET TM + TiO_2_” with different resolutions (**a**,**b**); cross-section (**c**), EDA-mapping by elements: carbon (**d**), oxygen (**e**), and titanium (**f**).

The photocatalytic activity of the systems was studied by the degree of decomposition of the model pollutant Rhodamine B in an aqueous solution (2.5 mg/L). For the photocatalytic tests, 3 g of Rhodamine B solution was inserted into the reactor representing the polypropylene Petri dish (20 mm in diameter) with the experimental sample of “PET TM + TiO_2_” system. The Petri dish was placed on the platform of IKA Vortex 4 digital with orbital shaking trajectory (IKA-Werke GmbH & Co. KG, Staufen im Breisgau, Germany) at 500 rpm to ensure the uniform mixing of the reaction solution and liquid access to the sample surface. Photocatalysis was provided under UV irradiation within 4 h. Mercury lamp 9 W with λ_max_ 365 nm (Cixi Jindan Electric Appliances Factory, Zhangqi industrial zone, Zhangqi, China) was used as a source and was located above the reactor at a distance of 3 cm (Figure 1). The emission spectrum of used mercury lamp is given on the insert in Figure 1. The second maximum at 404 nm (3.1 eV) has no significant influence on the photocatalytic process because of its low energy. The change in the concentration of the analyte in the solution was determined from the optical absorption spectra in the wavelength range of 400–800 nm. Spectra normalization was realized via the maximum intensity of absorption in the initial solution for all results. To determine the decomposition degree, reference curve in the concentration range from 0 to 10 mg/L was exploited (Figure 2). The curve area 0–2.5 mg/L was rebuilt according to the approximation equation with determined coefficient R^2^ = 0.9996 and then normalized again to 1 (Figure 2b). To use the calibration curves, each time, the absorption spectrum of the initial solution with the maximum concentration (2.5 mg/L in this case) was recorded, according to which the remaining measurements were normalized. The influence of dye absorption by the membrane on the absorption spectra of solutions was not instrumentally recorded as significant. The maximum registered absorption is about 5% after two hours of dark conditions; thus, we started the main experiment with UV exposure straight (Figure 3). All absorption spectra were registered at room temperature using the spectrophotometer MC 122M UVI-Vis (JSC “SOLAR”, Minsk, Belarus).

### 2.6. Antibacterial Properties Control

For the study of antibacterial activity, *Escherichia coli* (*E. coli*) and *Staphylococcus aureus* (*St. aureus*) were used as test cultures. Then, 0.6 mL of the culture liquid was placed on the sample of “PET TM + TiO_2_” systems, then the sample was irradiated with UV (365 nm, 0.01 mW/cm^2^) for 1 h while maintaining the temperature at 25 °C. Studies were carried out using bacterial colonies in the presence of “PET TM + TiO_2_” systems with and without a 5 × 5 cm size to take into account the effect of ultraviolet (UV) irradiation on bacterial colonies. The control sample was kept in the dark for an hour simultaneously. Then, 0.1 mL of washout from the samples were placed in a nutrient medium, then incubated in a thermostat for 48 h at a temperature of 30 °C. After cultivation, the number of colonies with typical morphological signs was determined, followed by recalculation of colony-forming units per 1 mL of solution (CFU/mL).

## 3. Results and Discussion

### 3.1. Morphology Control

As a base for preparing the breathable films with self-cleaning and antibacterial surfaces, ion-track PET membranes with pore diameters of 500 ± 15 nm were used. As a result of the two-stage hydrolysis of titanium dioxide, a film coating of TiO_2_ sintered nanoparticles uniformly distributed over the membrane surface was formed via the sol–gel method on the surface of the polymer membrane (Figure 3a–c).

A high-resolution SEM image of a modified membrane shows that TM is covered with a continuous layer and also has individual TiO_2_ nanoparticles with sizes up to 100 nm formed on the membrane surface (Figure 3b). TiO_2_ is localized not only on the PET TM surface but also on the pore walls. In this case, nanoparticles on pore walls have a smoother shape compared to nanoparticles on the surface. According to the cross-section of the PET TM (Figure 3c), the thickness of the titanium dioxide layer on the membrane surface is about 80 nm. EDA-mapping (Figure 3d–f) of the sample “PET TM + TiO_2_” surface demonstrates a uniform distribution of titanium dioxide over the surface of the PET membrane. As we previously reported [29], by changing the concentration of TiO_2_ in sol–gels, we were able to decrease/increase the amount of absorbed TiO_2_ nanoparticles, but the morphology of the function layer was changed from individual nanoparticles to their constant layer. The optimized concentration was found to be 0.5 wt.%.

### 3.2. Structural Analysis

As previously reported [29], XRD studies indicate the presence of TiO_2_-anatase on the surface of PET films in an amount of 23%.

**Figure 4:**
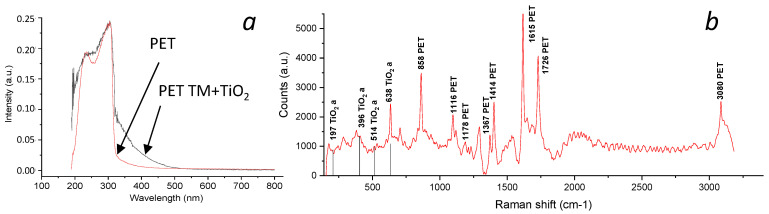
UV-vis spectra of initial PET TM and “PET TM + TiO_2_” (**a**) and Raman spectra of “PET TM + TiO_2_” systems (**b**).

The Raman spectrum of the “PET TM + TiO_2_” systems confirms the presence of TiO_2_-anatase on the surface of PET films (Figure 4b). The characteristic Raman bands of the anatase crystalline form are observed at 197, 396, 514, and 638 cm^−1^. Also, the Raman bands of PET were found. The 1615 cm^−1^ peak of PET, as already mentioned, corresponds to the Raman activity of the symmetric stretch of the skeletal 1,4-para substituted benzene rings. The peak at 1485 cm^−1^ in the Raman spectrum of polyethylene terephthalate (PET) is associated with vibrations of atoms in the aromatic ring structures of the polymer. This peak corresponds to the stretching vibrations of C-C bonds in benzene rings, which are the main structural elements of PET. The peak at 1294 cm^−1^ is due to vibrations of the C-C bond in aromatic rings and may be associated with vibrations of the COC group. The peak in the region of 1652 cm^−1^ is associated with vibrations of carbonyl groups (C=O), which are present in a part of the PET molecule. 1178 cm^−1^ peak of PET is associated with the vibrations of C–O–C bonds. Additionally, 1726 cm^−1^ peak of PET is associated with C=O. Peaks in 858, 1414, and 1367 cm^−1^ are substituted benzene rings, and peak in 3080 cm^−1^ is associated with C–H bonds in the methyl groups.

The UV-vis spectra of initial PET TM and “PET TM + TiO_2_” (Figure 4b) are consistent with the literature data [30]. In general, when a permanent layer of titanium dioxide is formed on the surface of the TM, the absorption region of the sample expands compared to the initial PET TM. The measurement of the absorption spectrum of TiO_2_ films is complicated with the superposition of a large number of transitions and corresponds to the anatase phase. Absorption is observed in the wavelength range of up to 400 nm. The absorption peaks can be attributed to the following: the peak at 340 nm to the amorphous phase (O 2p state) and the peak at 390 nm to the Ti 3d state. The adsorbed energy will be spent on the photocatalytic process of decomposition of an organic substance, an example of which can be the Rhodamine B dye taken in our work.

The air begins to pass through the membrane at an overpressure of 0.4 kPa; this is the first value that can be recorded using the instrument, and it can be assumed that the applied overpressure is lower than fixed.

Water passes through the membrane at an overpressure of 12 kPa (the minimum recorded value). The water permeability of “PET TM + TiO_2_” systems is shown in Table 1. The performance in this case is 3.057 mL/min*m^2^ (approximately one drop in 5 min). A drop left on the surface of a functionalized membrane, fixed horizontally, percolates within 4–5 min. It is possible to make a conclusion about the water permeability at a lower overpressure.

### 3.3. Evaluation of Applicability

#### 3.3.1. Surface Adsorption Properties Control

**Figure 5:**
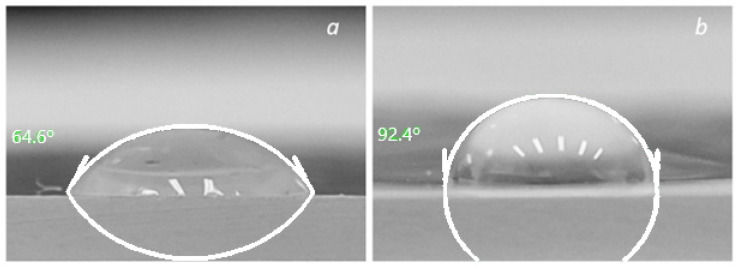
Wetting contact angle of PET TM (**a**) and “PET TM + TiO_2_” (**b**).

Considering that the main practical applications of breathable films with self-cleaning and antibacterial surfaces are associated with the use of liquid media, the changes in CA before and after the TiO_2_ functionalization of PET TM were checked. The original PET TM is characterized by a wetting CA of 64.6 ± 2.5 degrees (Figure 5) [31]. The nanostructured titanium dioxide coating modified the surface morphology and increased CA to 92.4 ± 3.2 degrees, which improved the hydrophobic properties of TM. CA more than 90 degrees of the “PET TM + TiO_2_” systems indicate weak hydrophobic properties.

#### 3.3.2. Analysis of Photocatalytic Activity

**Figure 6:**
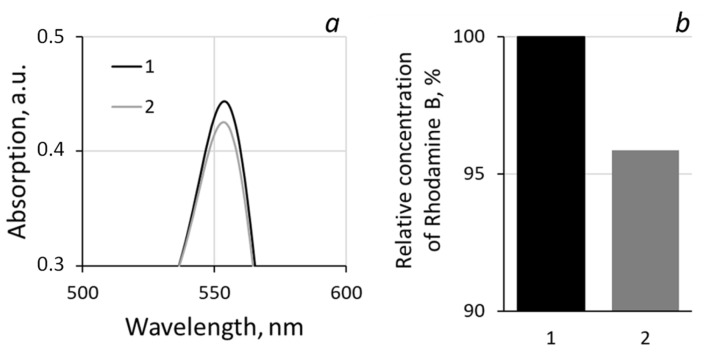
Evolution of the main absorption peak of Rhodamine B (**a**) before (1) and after (2) dark exposure for two hours (non-normalized spectra) and their percentage (**b**) with the presence of “PET TM + TiO_2_” system.

**Figure 7:**
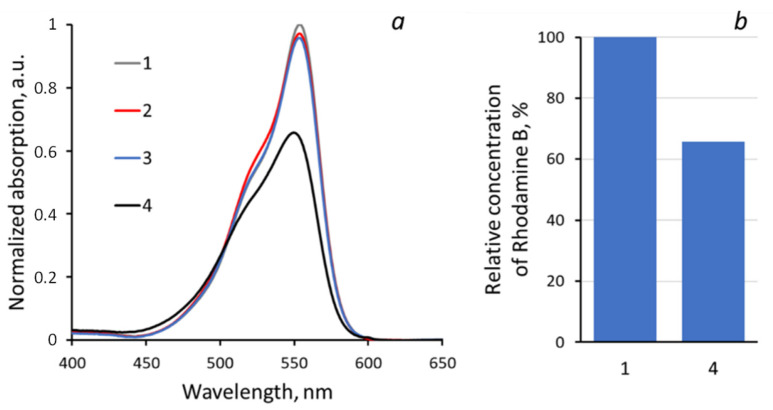
Absorption spectra (**a**) and relative concentration of Rhodamine B (**b**) of test solutions: 1—initial, 2—with “PET TM + TiO_2_” in the dark, 3—with “PET TM + TiO_2_” under the day light, 4—with “PET TM + TiO_2_” after UV exposure.

Figure 6 shows the evolution of the main absorption peak of Rhodamine B before (1) and after (2) dark exposure for two hours (non-normalized spectra). A slight degradation was evaluated. Figure 7 shows the absorption spectra of the initial test solution of Rhodamine B and the solution after exposure in the presence of “PET TM + TiO_2_” systems. To establish the absolute concentration of test dye, the normalized maxima of the absorption peak (at 554 nm) were correlated with calibration curves (see Figure 2b), and the results are collected in Table 2. The relative decrease in the Rhodamine B concentration in solution is about 34% for the exposure to the “PET TM + TiO_2_” system under UV irradiation. Furthermore, in the absorption spectra, the maximum shift from 554 nm of the initial solution to 550 nm of the solution after exposure with the shoulder at 522 nm smoothing is observed. It can be explained via the two processes of photodegradation of Rhodamine B, including the “classical photo mineralization” process leading to the complete destruction of the pollutant molecules and the accompanying process of the stepwise elimination of two diethylamino groups in the Rhodamine B molecules that do not affect the chromophore structure. The spectra evolution for the cases with “PET TM + TiO_2_” in the dark and under the daylight demonstrates the main role of the photocatalytic destruction of the dye molecules in the solution purification and not only due to the absorption via the membrane.

#### 3.3.3. Antibacterial Properties Control

**Figure 8:**
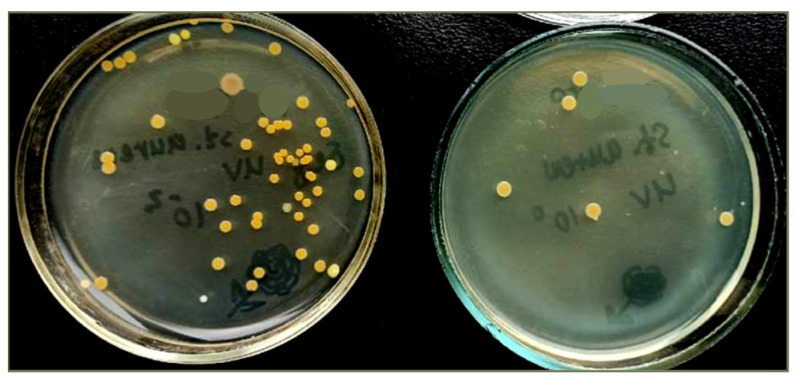
Antibacterial properties of film samples in relation to test bacteria *St. aureus* ATCC 6538 (without and with UV irradiation).

Membranes coated with titanium dioxide nanoparticles are capable of photocatalytic decomposition of organic compounds, including causing oxidative stress for bacteria, which, in turn, will affect their viability. The effect on bacterial viability was studied using *E. coli* and *St. aureus*. The logarithmic reduction factor was taken as critically significant for determining the antibacterial activity and was determined as follows:

*Lg Red* = *lg*(*C*/*C*_0_)

where *Lg Red*—logarithmic reduction factor; *C*_0_—cells concentration before the experiment; and *C*—concentration of bacterial cells after the experiment. In our case, *C* was considered in two versions: (1) *C*_TiO_2_+UV_—concentration of bacterial cells on the surface of “PET TM + TiO_2_” systems after UV-irradiation and (2) *C*_UV_—control the concentration of cells after UV-irradiation without “PET TM + TiO_2_” systems. The results are summarized in Table 3. Also, visual images of cells with the developed system are shown in Figure 8.

A decrease in colony count was recorded for both *E. coli* and *St. aureus* strains and recorded when irradiated with UV. However, when these strains were irradiated in the presence of “PET TM + TiO_2_” systems, a significant decrease in the concentration of live bacteria was established. It indicates that “PET TM + TiO_2_” systems exhibit antibacterial activity against the *E. coli* and *St. aureus*; the logarithmic reduction factors are 3.34 and 4.24, respectively.

Our PET TM + TiO_2_” systems use two mechanisms that contribute to the antibacterial effect. The most significant contribution is made using the photocatalytic activity of TiO_2_. When exposed to UV radiation on photocatalytically active substances, free radicals (reactive oxygen species, ROS) are produced in the biological environment, which causes lipid peroxidation, damage to membranes, and damage to the structure of DNA and organelles of microorganisms. This is due to the transition under the action of light of a valence electron to the conduction band of the photocatalytic material, as a result of which ROS are also formed, mainly hydroxyl radicals (•OH), which play the role of holes in an aqueous medium and lead to the oxidation of biological molecules. The main feature of our PET TM + TiO_2_” systems is weak hydrophobicity due to which, in combination with photocatalytic activity, small colonies of bacteria that can still develop (according to the results) on the surface of the systems during tests in a nutrient medium, under real conditions, will not be able to attach to the surface.

It should be noted that at the moment, “PET TM + TiO_2_” systems do not demonstrate outstanding results in terms of self-cleaning and antibacterial properties. The main problem of “PET TM + TiO_2_” systems is that the main part of absorption spectra is λ <400 nm, which complicates the use of such systems by exposing them to natural sunlight. Further work on optimizing the “PET TM + TiO_2_” systems will be aimed at the shift of TiO_2_ absorption into the red zone via the changes in coating modes or post-processing of the resulting systems, for example, as shown in our previous work [29], for expansion of the prospects of practical use. However, the potential of using breathable films with self-cleaning and antibacterial surfaces based on PET membranes coated with titanium dioxide for various purposes, for example, for packaging food products or sterile medical plasters is already obvious.

## 4. Conclusions

The potential for developing breathable films with self-cleaning and antibacterial surfaces based on TiO_2_-functionalized PET membranes via the sol–gel method is evident. The application of this technique yielded a uniform TiO_2_ coating composed of particles with characteristic sizes less than 100 nm, and an anatase content of 26% was successfully achieved.

To illustrate the photocatalytic activity of the “PET TM + TiO_2_” systems, the degradation of Rhodamine B was used as a demonstrative example. The results displayed a photocatalytic effect, as evidenced by a relative 31% decrease in Rhodamine B concentration in 4 h, corresponding to the reduction in absorption at 455 nm.

Moreover, the “PET TM + TiO_2_” systems exhibited notable antibacterial properties, as they displayed substantial logarithmic reduction factors of 3.34 against *E. coli* and 4.24 against *St. aureus*, respectively. This indicates their potential as effective agents for combatting bacterial growth, making them highly desirable for various applications.

With their inherent antibacterial and self-cleaning capabilities, these systems hold significant promise in the creation of active functional materials. They are particularly well-suited for producing breathable films with self-cleaning and antibacterial surfaces, making them ideal candidates for applications such as food packaging or sterile medical plasters. Notably, one of the major advantages of the developed “PET TM + TiO_2_” systems is their capacity for multiple reuses, enhancing their overall sustainability and cost-effectiveness.

## Figures and Tables

**Table 1:** Water permeability of “PET TM + TiO_2_” systems.

Overpressure, kPa	Performance, mL/min*m^2^
0	0
12	3.057
20	15.286
40	152
60	713
80	815
120	917

**Table 2:** Concentrations of Rhodamine B in test solution according to the calibration curve (Figure 2b).

Spectra (Figure 7)	Normalized Absorption, a.u.	Concentrarion of Rhodamine B, mg/L	Decrease in Concentration, mg/L	Decrease in Concentration, %
1	1	2500	--	--
2	0.971	2431	0.069	2.76
3	0.959	2399	0.101	4.04
4	0.658	1654	0.846	33.84

**Table 3:** The results of the antibacterial activity investigation.

Test Bacteria	Concentration of Bacterial Cells, CFU/mL			*Lg Red*	
	*C*_0_	Experiment *C*_TiO_2_+UV_	Control *C*_UV_	“PET TM + TiO_2_” + UV	UV
*E. coli*	5.0 × 10^5^	2.3 × 10^2^	2.2 × 10^5^	3.34	0.36
*St. aureus*	8.6 × 10^5^	5.0 × 10^1^	4.7 × 10^4^	4.24	1.26

## Data Availability

Not applicable.
